# Custom Made Replacement of the Mandibular Condyle in a Case of Fibrous Dysplasia with Cystic Degeneration; A Case Report

**DOI:** 10.3390/dj4040042

**Published:** 2016-11-15

**Authors:** Jorinde S. L. I. Oostenbroek-Bisschop, Jop P. Verweij, J. P. Richard van Merkesteyn

**Affiliations:** Department of Oral and Maxillofacial Surgery & Special Dental Care, Leiden University Medical Centre, 2333 ZA Leiden, The Netherlands; J.S.L.I.Oostenbroek-Bisschop@lumc.nl (J.S.L.I.O.-B.); J.P.Verweij@lumc.nl (J.P.V.)

**Keywords:** fibrous dysplasia, fibro-osseous, osseous, bone, TMJ, temporomandibular joint

## Abstract

This paper describes a rare case of fibrous dysplasia with cystic degeneration in the mandibular condyle. Diagnostic and therapeutic considerations are discussed. A 40-year old woman presented with pain near the region of her right ear. Physical and radiographic examination showed no abnormalities besides the presence of a mixed radiopaque/radiolucent expansive lesion of the right condyle. Pathologic examination showed high bone-turnover with bone formation. Bone scintigraphy showed a monostotic active fibrous lesion in the right part of the mandible. Bisphosphonate treatment did not sufficiently treat the patient’s symptoms and physiotherapy to treat craniomandibular dysfunction as a factor in the pain was also unsuccessful. The patient later developed an acute external otitis due to a narrowed outer ear canal and had to be admitted to the hospital for treatment with intravenous antibiotics. Approximately two years after first presentation, resection of the affected bone (condylectomy) and reconstruction with a custom total joint prosthesis was indicated due to repeated functional deficits with considerable morbidity. Pathologic examination of the resected mandibular condyle showed increased bone formation including formation of neocortex and some cystic formation. This was diagnosed as fibrous dysplasia with cystic degeneration. Approximately two years after surgery, the patient functioned well.

## 1. Introduction

Fibro-osseous lesions of the jaws are a diverse group of neoplasms, wherein bone is replaced by cellular fibrous connective tissue [1]. Although most fibro-osseous neoplasms are benign, with only a very small chance of malignant transformation, these expansive tumours can be locally invasive and cause significant morbidity [1,2]. Fibrous dysplasia is probably the most well-known fibro-osseous lesion. This intrinsic bone lesion usually presents as a progressively expansive lesion with a radiopaque aspect on the X-ray image, sometimes in combination with radiolucent aspects [3]. Fibrous dysplasia is however part of a broad clinical spectrum and can therefore present in many different forms [3,4]. Formation of cysts (cystic degeneration) can occur following fibrous dysplasia, but reactive bone formation following expansion of a bone cyst shows similar characteristics and should be considered as an alternative diagnosis in these patients [5].

The presence of fibro-osseous lesions or bone cysts in the mandibular condyle is rare. When an expansive neoplasm is present however, the risk of functional problems is high because of the complex function of the temporomandibular joint. In these patients, treatment is thus often indicated. In complex cases, surgical therapy in the form of a complete condylectomy can be necessary. Subsequent reconstruction of the temporomandibular joint is aimed at restoration of function. Widely used techniques are condylar reconstruction, with an autologous bone graft (for example costochondral reconstruction); or reconstruction with an allogeneic custom total joint prosthesis [6].

This study reports a rare case of fibrous dysplasia with cystic degeneration, that was treated with resection of the mandibular condyle and reconstruction with a custom total joint prosthesis. The characteristics of fibrous dysplasia, differential diagnoses, and treatment plan in this case are discussed.

## 2. Case Report

**Figure 1:**
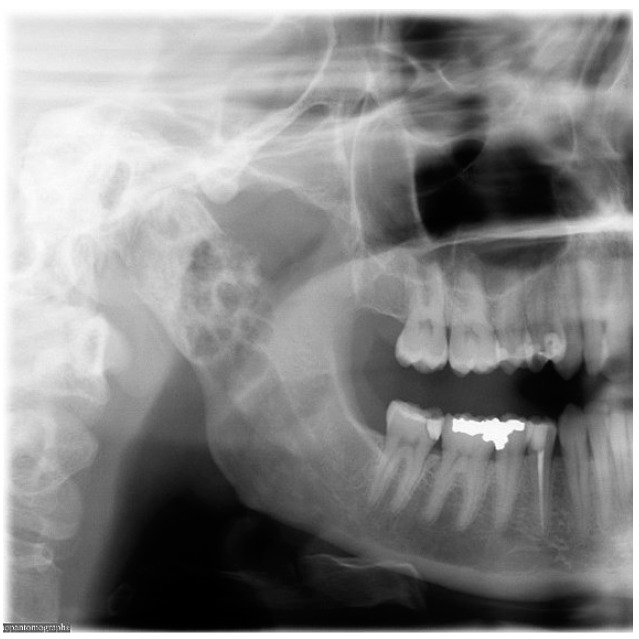
Orthopantomograph of the patient during first presentation showing a mixed radiopaque/radiolucent expansive lesion of the right mandibular condyle.

A healthy, 40-year old woman presented to the emergency room with pain near the region of her right ear. She had furthermore experienced vertigo with nausea for several months. Physical and radiographic examination by the neurologist and Ear-Nose-Throat-surgeon showed no abnormalities at the time of presentation. The only abnormal finding at this time, however, was enlargement of the right condyle and the patient was therefore referred to the maxillofacial surgeon (Figure 1).

**Figure 2:**
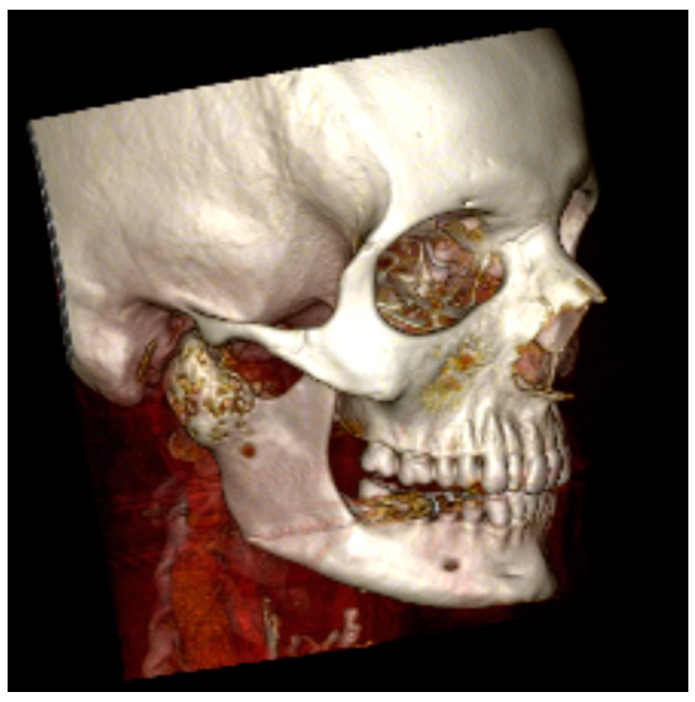
Computed tomography of the peroperative presentation, showing an expansive lesion of the right mandibular condyle.

Computed tomography (CT) showed a relatively large (34 × 30 × 28 mm) bone tumour in the right condyle with cystic deformation without involvement of the inferior alveolar nerve or other surrounding structures (Figure 2). Magnetic resonance imaging (MRI) showed an expansive lesion of the right condyle with heterogeneous signal intensity on all recordings (T1, T2, and after intravenous contrast). The tumour in the right condyle caused compression of the parotid gland but did not show invasive growth into this gland or other surroundings structures. The differential diagnoses for this bone tumour included giant cell tumour, aneurysmatic bone cyst, ameloblastoma, fibrous dysplasia, and myxoma. A biopsy of the lesion was performed under general anaesthesia.

Microscopic examination by a specialized pathologist showed mainly pre-existing bone with a high bone turnover and bone formation. Fibrous dysplasia was deemed most likely, but a certain diagnosis was not possible. The biopsy showed no signs of malignancy.

**Figure 3:**
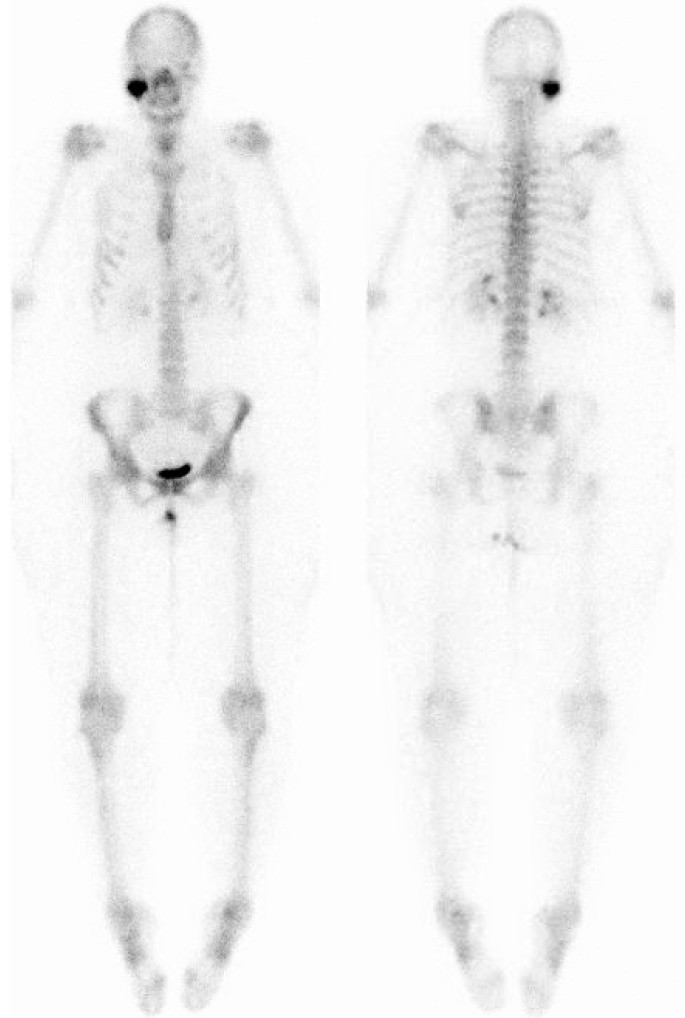
Bone scintigraphy showing a monostotic lesion in the right mandibular condyle.

Approximately four months after initial presentation at the emergency room, the patient was referred to a maxillofacial surgeon who specialized in bone tumours. Physical examination showed a narrow outer ear canal on the right side without signs of infection at the time of inspection. The masseteric and pterygoid muscles were hypertrophic and painful during palpation. The maximum mouth opening was 13 mm with a deviation to the right side and a reduced laterotrusive movement of 4 mm to the right side (compared to 10 mm to the left side). Computed tomography showed a stable situation compared to the CT-scan that was taken four months previously. Bone scintigraphy showed a monostotic active fibrous distension in the right part of the mandible (Figure 3).

Bisphosphonate treatment was commenced with olpadronate, 100 mg daily. The patient was furthermore referred to a physiotherapist to treat the symptoms of craniomandibular dysfunction. Besides an increase of the maximum mouth opening to 17 mm, this however did not have the desired effect. Bisphosphonate treatment did somewhat relieve the pain, but the patient still experienced high morbidity due to the remaining pain symptoms. During follow-up, the patient experienced infectious symptoms around the right ear twice, which was diagnosed as otitis of the outer ear canal due to compression and subsequent narrowing of the canal. The patient had to be admitted for four days and received intravenous antibiotics to reduce the infection.

Because of the repeated functional deficits with considerable morbidity for the patient, surgical therapy was therefore indicated. After consultation with several specialists in the field, resection of the affected bone (condylectomy) and reconstruction with either rib bone or a custom total joint were believed to be the two best options. Total joint replacement with a custom total joint was preferred by the surgeon and planned approximately two years after the first presentation at the emergency room.

The patient was preoperatively scanned in her intended occlusion to fabricate the custom total joint. A stereolithographic model of the jaw was printed to define the extent of the pathology and the margins for resection. Because the custom joint contains nickel, the patient was tested for nickel allergy, which was not present.

**Figure 4:**
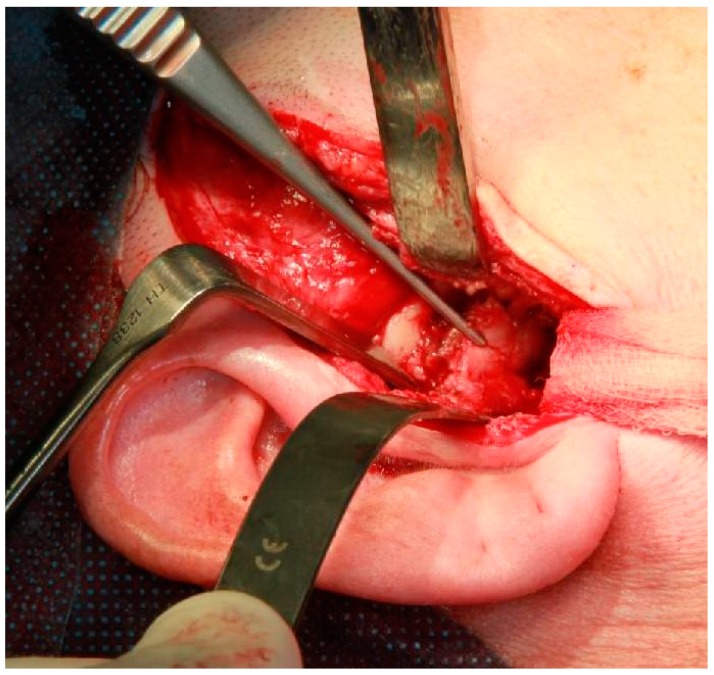
Intraoperative photograph of excision of the right mandibular condyle via an extra-oral approach.

Under general anaesthesia, the condyle was resected through a pre-auricular and submandibular approach (Figure 4). A custom made resection template was used to define the margins. After resection, a custom-made Biomet temporomandibular joint prosthesis, including the condylar head and articular fossa was inserted and fixated with titanium screws.

**Figure 5:**
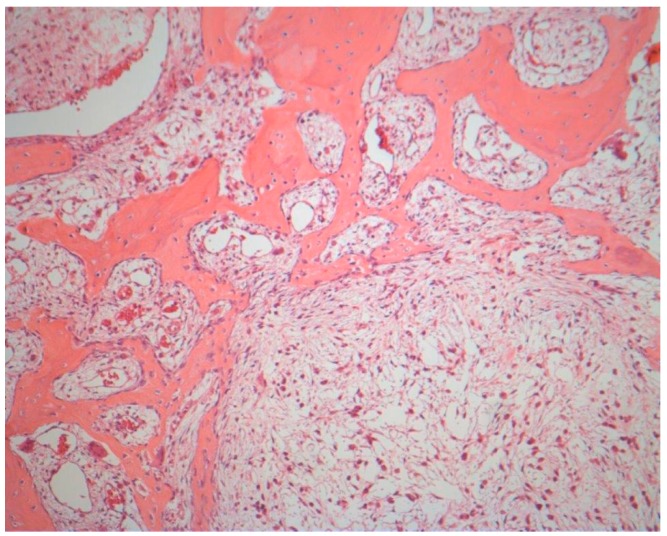
Microscopic image showing increased bone turnover with some cystic formations. This was diagnosed as fibrous dysplasia with cystic degeneration.

Pathologic examination of the resected tissue showed a predominantly expansive lesion with some cystic formations, highly increased bone turnover, and reactive/pre-existing bone formation including formation of neocortex. This was diagnosed as fibrous dysplasia with cystic degeneration (Figure 5).

**Figure 6:**
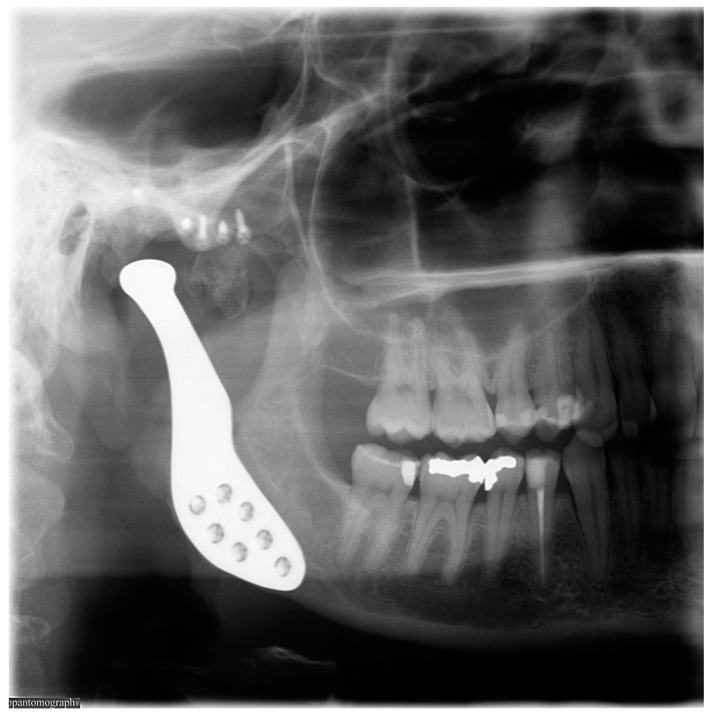
Postoperative orthopantomograph showing a custom total joint replacement.

After uneventful healing, the patient was treated by the gnathologist and physiotherapist to help retrieve complete masticatory function. Approximately 5 months after surgery, the patient reported complete function without any pain or morbidity. The occlusion showed no changes. The maximum mouth opening was 25 mm after treatment with Therabite. During the latest follow-up, approximately 22 months after surgery, the patient was free of complaints and satisfied with the result (Figure 6).

## 3. Discussion

This case reports a rare case of fibrous dysplasia of the right mandibular condyle that was treated with condylectomy and a custom total joint prosthesis. This paper shows that fibrous dysplasia can occur even in the mandibular condyles and treatment can be necessary because of complaints. In this particular case, the disease manifested as part of the spectrum that is fibrous dysplasia, also showing signs of cystic degeneration and both reactive and pre-existing bone formation.

Fibro-osseous lesions of the jaws are classically categorized based on their either developmental, reactive, or neoplastic origin [7]. The correct diagnosis depends on their clinical, radiological, and histopathologic features. This case however shows that the classification of fibro-osseous subtypes (and more specifically the diagnosis of fibrous dysplasia) is not always that clear.

*Clinically*, craniofacial fibrous dysplasia usually presents as a slowly growing, benign bone lesion. Patients are usually around 25 years old and present with asymmetry, swelling and/or pain [4]. In some cases, the lesion is however discovered during routine (radiographic) examination at a later age. Fibrous dysplasia is categorized as monostotic or polyostotic. After diagnosing a patient with fibrous dysplasia, bone scintigraphy, to expose possible other lesions of fibrous dysplasia lesions, is therefore always indicated [8]. In the current case, only the right mandibular condyle was affected and the patient was thus diagnosed with monostotic fibrous dysplasia.

*Radiographically*, craniofacial fibrous dysplasia usually shows as a relatively well-defined mixed radiopaque and radiolucent expansive lesion [9]. Computed tomography shows an expansive lesion with a ‘ground glass’ appearance [3,10]. Radiolucency of the lesion varies with the amount and degree of mineralization [11]. Especially in older individuals, craniofacial fibrous dysplasia may appear more heterogeneous with focal cystic and sclerotic areas [3].

*Histopathologically*, fibrous dysplasia is believed to arise from abnormal activity in the bone forming mesenchymal tissue. It is part of the spectrum of benign fibro-osseous lesions, characterized by replacement of normal bone with a fibrous connective tissue that gradually undergoes mineralization and shows varying degrees of osseous metaplasia [2,7]. Microscopic images characteristically show replacement of normal bone with a cellular fibroblastic stroma. Irregular bone trabeculae composed of immature woven bone that evolves directly from the stroma are typically present [1]. Mutation of the GNAS-1 gene (located on chromosome 20) can be evaluated if tissue is cryopreserved after excision. Among fibro-osseous lesions, this mutation is specific for fibrous dysplasia, although sensitivity is low [12]. In our patient, no GNAS mutation was present.

The differential diagnosis in the current patient included aneurysmal bone cyst, simple bone cyst, ameloblastoma, giant cell tumor, chondroblastoma, osteochondroma, and myxoma.

An aneurysmal bone cyst (ABC) is an expansive osteolytic lesion consisting of blood-filled spaces and channels divided by connective tissue septa that contain osteoid-like tissue [13]. An ABC usually either presents asymptomatic or with acute onset of expansion and localized pain [3]. In the current literature, this rare condition has been reported in the mandibular condyle less than twenty times [13]. An association between an ABC and fibrous dysplasia has been reported, wherein the exact relation remains unclear. One case report on an ABC of the mandibular condyle suggested conversion of an ABC into fibrous dysplasia, but acknowledged that the simultaneous presence of both conditions can also occur [14]. Another author, on the other hand, reported on cystic degeneration of cranial fibrous dysplasia causing rapid enlargement of the fibrous dysplastic lesion [15]. In our patient, cystic degeneration was present and an ABC was considered. The patient’s age and histopathologic findings did however not correspond with an aneurysmal bone cyst, and it was therefore not the likely diagnosis in our patient.

A simple bone cyst of the mandibular condyle has been reported less than 10 times in the current literature [16]. It is a usually an asymptomatic radiolucent lesion, often with a thin radiopaque line [17]. Expansion of the cortical plates and reactive bone formation can be observed. Therefore, two diagnostic possibilities were considered in our patient: (1) a pre-existing cystic process with reactive bone formation; or (2) a pre-existing fibro-osseous lesion with cystic degeneration. In the current case, there was extensive bone formation with several histopathologic characteristics of fibrous dysplasia. Therefore, a pre-existing fibro-osseous lesion (fibrous dysplasia) with cystic degeneration was deemed the most likely diagnosis.

Ameloblastoma is a benign, locally invasive, cystic tumour of the jaws. It typically presents in the region of the mandibular molars and ramus and does not often occur in the condyle [18,19]. Radiographically, ameloblastomas present as either a well-demarcated unilocular (unicystic) or multilocular (multicystic) radiolucent lesion of the jaw [20]. Multicystic ameloblastomas can show a ‘soap-bubble shape’. Ameloblastomas show considerable variation in microscopic appearance [20]. Histologic evaluation typically shows proliferation of epithelial cells that impress as ameloblasts.

Giant cell tumour, chondroblastoma, osteochondroma and myxoma are benign (but locally destructive) tumours that very rarely occur in the mandibular condyle [21,22,23,24]. These tumours radiographically all present as relatively well-defined osteolytic lesions. In our current patient, the clinical and radiographic presentation with extensive bone formation, as well as the histopathologic examination, did not correlate with these diagnoses.

Total temporomandibular joint replacement is evidently a last resort in condylar surgery. In our patient, treatment was necessary because the patient experienced pain and the large expansive tumour caused otitis that even required admittance to the hospital and intravenous antibiotic therapy. Treatment with bisphosphonates aimed to treat the symptoms and reduce expansion of the tumour did not have enough effect. Therefore, resection of the temporomandibular joint was the only predictable treatment option.

After resection of the mandibular condyle, either reconstruction with an autogenous bone graft, for example a costochondral graft, or reconstruction with an alloplastic custom total joint prosthesis are both good treatment options. Especially in young individuals, the autogenous bone graft is preferred, while in adults, alloplastic reconstruction with a custom total joint prosthesis is also a safe and predictable treatment option [6]. Custom total joint prosthesis have shown to provide a stable, improved long-term outcome resulting in quality of life improvement [25]. Because the TMJ is placed under heavy cyclical loading and unloading, custom prosthesis are preferable over stock devices [26].

In this case, our patient functioned well and was free of complaints two years after reconstruction with a custom alloplastic total joints prosthesis.

## 4. Conclusions

This paper illustrates a rare presentation of fibrous dysplasia with cystic degeneration, and reports the subsequent treatment including rehabilitation of the temporomandibular joint and of the occlusion.
